# Pediatric falls from windows and balconies: incidents and risk factors as reported by newspapers in the United Arab Emirates

**DOI:** 10.1186/s13017-017-0156-z

**Published:** 2017-10-16

**Authors:** Michal Grivna, Hanan M. Al-Marzouqi, Maryam R. Al-Ali, Nada N. Al-Saadi, Fikri M. Abu-Zidan

**Affiliations:** 1 Institute of Public Health, College of Medicine and Health Sciences, UAE University, Al-Ain, United Arab Emirates; 2 Medical Student, College of Medicine and Health Sciences, UAE University, Al-Ain, United Arab Emirates; 3 Department of Surgery, College of Medicine and Health Sciences, UAE University, Al-Ain, United Arab Emirates

**Keywords:** Falls from windows, Balconies, Children, UAE

## Abstract

**Background:**

Falls of children from heights (balconies and windows) usually result in severe injuries and death. Details on child falls from heights in the United Arab Emirates (UAE) are not easily accessible. Our aim was to assess the incidents, personal, and environmental risk factors for pediatric falls from windows/balconies using newspaper clippings.

**Methods:**

We used a retrospective study design to electronically assess all major UAE national Arabic and English newspapers for reports of unintentional child falls from windows and balconies during 2005–2016. A structured data collection form was developed to collect information. Data were entered into an Excel sheet and descriptive analysis was performed.

**Results:**

Newspaper clippings documented 96 fall incidents. After cleaning the data and excluding duplicate cases and intentional injuries, 81 cases were included into the final analysis. Fifty-three percent (*n* = 42) were boys. The mean (range) age was 4.9 years (1–15). Thirty-eight (47%) children fell from windows and 36 (44%) from balconies. Twenty-two (27%) children climbed on the furniture placed on a balcony or close to a window. Twenty-five (31%) children were not alone in the apartment when they fell. Twenty-nine children fell from less than 5 floors (37%), 33 from 5 to 10 floors (42%) and 16 from more than 10 floors (21%)**.** Fifteen children (19%) were hospitalized and survived the fall incident, while 66 died (81%).

**Conclusions:**

Newspapers proved to be useful to study pediatric falls from heights. It is necessary to improve window safety by installing window guards and raising awareness.

## Background

Unintentional falls are the second leading cause of injury-related hospitalization for all ages accounting for about 30% of injury admissions and 15% of all Emergency Department visits [1, 2]. Falls of children from heights (balconies and windows) often result in severe injuries and death [3]. These falls were described in a chapter called “Falling out of a window” in “The Book of Accidents” 1830. The authors stressed the importance of supervision and vigilance of parents and maids [4]. Community education and installation of window guards, starting in 1970s in several US cities, led to successful decrease of these injuries [3]. The famous intervention “Children Cannot Fly” targeting parents with extensive educational campaign and distribution of free window guards in New York City in 1976 resulted in 96% reduction of unintentional window falls [5, 6].

United Arab Emirates is a Middle-East country with a fast economic development characterized by diversification from the oil industry to other sectors, as tourism, retail and manufacturing. The population of the UAE increased rapidly and recently reached over 9 million inhabitants, of whom less than 18% are UAE Nationals [7]. Children less than 15 years old constitute about 20% of the population [7]. The country is a federation of seven emirates (Abu Dhabi, Ajman, Dubai, Fujairah, Ras Al Khaimah, Sharjah, and Umm al-Quwain).

Despite improvements in the health care, injuries remain a leading cause of morbidity and mortality in the UAE, especially among children and youth [8]. The injury death rate for children under 15 years old was 13.6 per 100.000 person-years during 2000–2008 [8]. Various ethnic groups with diverse socio-cultural, religious and educational background pose a special challenge for safety promotion in the UAE [8].

Details on child falls from heights in the United Arab Emirates (UAE) are not easily accessible. Our aim was to assess the incidents, activities and risk factors for pediatric falls from windows/balconies in the UAE using newspaper clippings.

## Methods

We used a retrospective survey to assess eight UAE national Arabic and English newspapers for reports on unintentional child falls from heights (windows, balconies) at residential buildings during 2005–2016. Children 0–15 years were included. Intentional injuries, as suicide, homicide, and from other buildings (school, hotel) were excluded. We searched newspapers electronically using key words including child/boy/girl/baby/toddler and fall/fell/died and window/balcony/height. A structured data collection form was designed.

Variables collected included demography, location of injury (Emirate), supervision of a child, equipment (furniture), environmental factors (balcony or window, number of floors) and outcome (died on spot, died in the hospital, survived). Nationality was divided into four categories (UAE national, Asian, other Arabs, and others). Four investigators did the search independently checking for completeness of reporting. Data of fall incidents were entered into an Excel sheet. Descriptive analysis was performed. Official letters were written to health authorities (Ministry of Health, Health Authority Abu Dhabi, Dubai Health Authority) in order to obtain mortality and morbidity reports on falls. The websites of health authorities were also checked. We searched also for information about safety policy and interventions.

Fisher’s exact test was used to compare categorical data of two or more independent groups. A *p* value of less than 0.05 was accepted as significant. Data were analyzed using Statistical Package for the Social Sciences (IBM-SPSS version 23.0, Chicago, Il, USA).

## Results

**Fig. 1:**
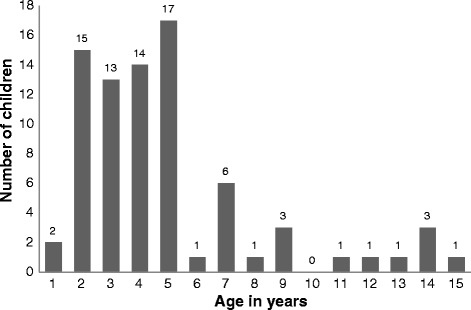
Pediatric falls from windows/balconies by age (*n* = 81)

Data from health authorities lacked details on personal and environmental risk factors and could not be studied. Newspaper clippings documented 96 fall incidents of children 0–15 years during the study period. After cleaning the data and excluding duplicate cases (3 cases), non-residential cases (1 at hotel, 1 at the airport, 1 at school) and intentional injuries (5 suicides; 3 homicides; 1 child was thrown from window during a fire by a mother), 81 cases were included in the final analysis. Fifty-three percent (*n* = 42) were boys and 4% (*n* = 3/75) were UAE-nationals. Male to female ratio was 1:1.1. The mean (range) age was 4.9 years (1–15) (Fig. 1, Table 1). Forty-nine percent (*n* = 39) were from the Emirate of Sharjah. Thirty-eight (47%) children fell from windows, 36 (44%) from balconies, and 7 (9%) cases were unknown. Twenty-two (27%) children climbed on the furniture placed on a balcony or close to a window.

**Table 1:** Demographic variables

Variable		*n*	%
Gender			
	Male	42	52.5
	Female	38	47.5
Age group			
	0–5	61	77.2
	6–10	11	13.9
	11–15	7	8.9
Nationality			
	UAE	3	4
	Asian	24	32
	Other Arab	40	53.3
	Other	8	10.7
Emirate			
	Sharjah	39	48.7
	Abu Dhabi	16	20
	Dubai	10	12.5
	Ajman	9	11.3
	Fujeirah	4	5
	RAK	2	2.5
Supervision			
	Yes	25	54.3
	No	21	45.7

*UAE* United Arab Emirates, *RAK* Ras Al Khaimah

Information about supervision was available in 43 cases (53%). There was another person present in the apartment when the child fell in 25 cases (31%) mother, father, or both parents in 18/25 cases (72%), grandmother or aunt in 2 cases, maid in 2 cases and older sibling in one case). Children were alone in 21 cases. One child was autistic.

**Fig. 2:**
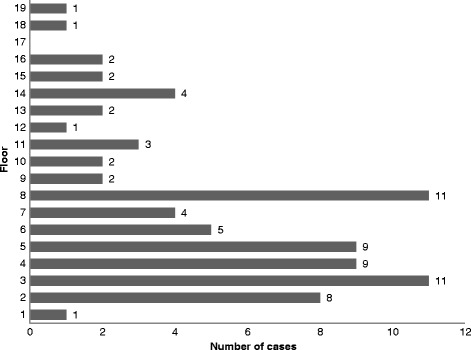
Pediatric falls from windows/balconies by floor (*n* = 78/81)

**Fig. 3:**
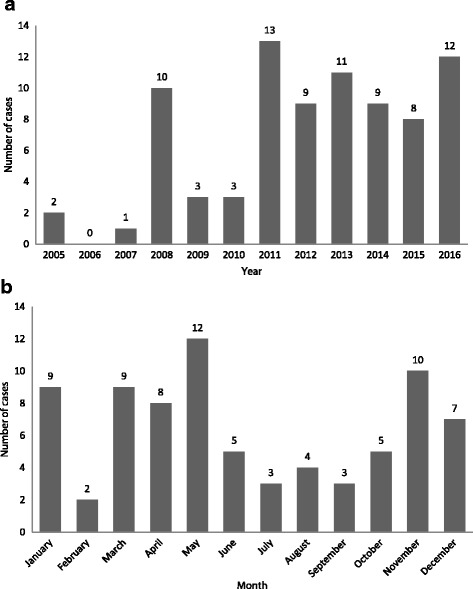
Pediatric falls from windows/balconies by year (a) and month (b) (*n* = 77/81)

**Fig. 4:**
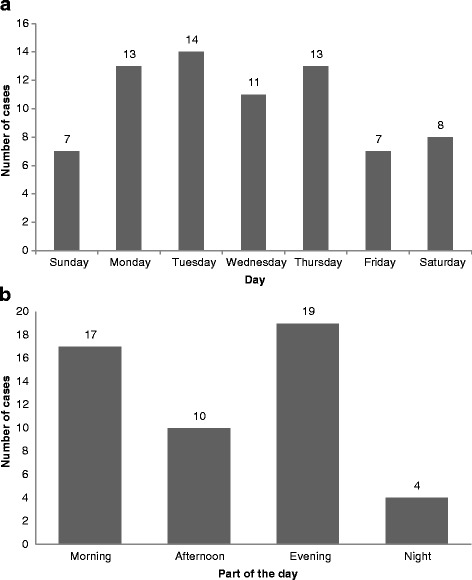
Pediatric falls from windows/balconies by day of the week (a) and part of the day (b) (*n* = 77/81; 24/81)

Twenty-nine children fell from less than 5 floors (37%), 33 from 5 to 10 floors (42%), and 16 from more than 10 floors (21%) (Fig. 2). Falls were more frequent in May (*n* = 12; 15%) (Fig. 3), in the evening (*n* = 19; 38%), and on Tuesday (*n* = 14; 19%), (Fig. 4). Forty-nine children died on spot (60%), 11 children died in the hospital (13%), and 15 (19%) were hospitalized and survived the fall incident. The place of death was unknown in six children (7%). 6/8 (75%) children falling from 1 to 2 floors survived, 7 out of 26 (26.9%) falling from 3 to 5 floors survived and 2/38 (5.3%) falling from more than 5 floor survived (*p* < 0.001, Fisher’s Exact test). We identified local governmental efforts to introduce new building regulations and increase public awareness. Changes in regulations were usually triggered by fatal fall incidents.

## Discussion

Children under 5 years old and those living in Sharjah Emirate were at high risk of falling from windows or balconies. Majority of those who fell from higher levels died. Many children were not alone in the apartments when they fell.

The male preponderance has been described in many studies [3, 9–11]. In contrast, our study showed a gender male/female ratio of 1:1.1. UAE-nationals were only 4% of all victims. UAE nationals usually live in villas having less number of floors with better supervision by maids. Similar to others, majority of our cases were children under 5 years of age [3, 9, 12]. Small actively moving children who love to explore things without appreciation of risks are more vulnerable to injuries [13]. A small body of a child can easily squeeze through small window gaps or railings of balconies.

The high incidence of falls in Sharjah Emirate can be explained by the high proportion of high buildings, and residents having low socio-economic status. If both parents are working and cannot afford to appoint a maid or if the mother is busy working at home, then there is lack of supervision of children.

Falls are more serious if they are from higher levels [12, 14, 15]. Children who fell from less than two floors in our study had a better chance to survive. Furthermore, kinetic energy absorbing surfaces, such as grass or vegetation may reduce the fall impact [15].

Fall incidents occurred more often in cooler months when there was no need to use air conditioning because families tend to open windows and balconies at that time. In other countries, there were more fall cases during the hot summer, especially among families with low-income living in buildings without air-conditioning [3, 10, 16]. Less falls occurred during the summer in our study. The exceptionally hot weather in our setting discourages opening the windows because of the air-conditioned environment. Furthermore, many families travel overseas for summer holidays.

More incidents occurred during the working days of the week and decreased during the weekends, possibly due to increased supervision by parents and older siblings. The time of incidents differs in the literature. Some studies showed more falls in the afternoon [3, 5], while others in the evening [9].

The information about supervision in newspapers was limited. Nevertheless, many of the children were not alone during the incident similar to a US study, in which more than half the falls occurred when a parent was at home [3]. In another study from Switzerland, the supervising person did not see the fall of the child. The child was left alone at home or left unattended for a short period of time [11]. Parents, maids, or siblings may have difficulty to watch children all the time. They may be distracted by other activities. Many parents undermine the importance of limiting access of children to balconies or windows. One alarming case study occurred when police saved a child sitting at the kitchen window by breaking into the apartment. The police warned the parents. Nevertheless, the same child fell to death from the balcony later on [17].

Our data suggest that furniture placed near a window or at a balcony is a contributing risk factor for falls, because the child has an easier access to the window. This was reported by others [3, 11, 16].

The government, municipalities, police, and building construction sectors in the UAE reacted to the fall incidents and have made active efforts to reduce the burden of falls over the last few years. This included introduction of new building and construction laws, enforcement of window guards, requiring minimum heights for railing at balconies, and educational campaigns. The challenge remains with the high turn-over of working expatriate families having different educational, cultural and socio-economic background, and various languages. The broad base public education should not focus only on parents, but also on maids, building owners, and managers [15].

### Limitations of the study

We have to acknowledge that there are certain limitations of our study. There is debate about the value of using newspaper clippings for injury prevention [18–20]. Despite that debate, we can observe the high precision of information on the floor level from which children fell and their associated mortality. We could not previously reach that level of accuracy in our prospectively collected data of a trauma registry [21]. We have previously studied charts of pediatric injured patients who fell from height and found that detailed information on risk factors was missing [22]. Furthermore, 60% of our cases in the present study died on scene having no hospital charts.

Available data reports on mortality and morbidity is usually lacking details on personal and environmental risk factors which can affect appropriate local prevention strategies. There is no unified health information system in the United Arab Emirates. Every health authority has its own injury data collection.

Although newspaper clippings contained rich information, some cases could have been missed, including less serious cases which may be treated in emergency rooms or hospitals. There is a possibility for selection bias by newspapers as they capture more serious conditions, while milder cases may have survived. Our study population represents only the tip of an iceberg. The exact time of incidents is lacking in our study. We could not also verify the reported information with official death reports. Although death reports in our setting improved by introducing an electronic reporting system, the access to data is limited. Some of our unintentional falls could be cases of child abuse or may include suicide attempts, especially among older children.

## Conclusions

Newspaper clippings proved to be useful to study national pediatric falls from heights. It is necessary to improve window safety by installing window guards and raising awareness among parents, maids, and building owners and managers.
